# Synthesis of Silver Nanoparticles Dispersed in Various Aqueous Media Using Laser Ablation

**DOI:** 10.1155/2014/324921

**Published:** 2014-09-08

**Authors:** M. Tajdidzadeh, B. Z. Azmi, W. Mahmood M. Yunus, Z. Abidin Talib, A. R. Sadrolhosseini, K. Karimzadeh, S. A. Gene, M. Dorraj

**Affiliations:** ^1^Department of Physics, Faculty of Science, Universiti Putra Malaysia (UPM), 43400 Serdang, Malaysia; ^2^Materials Synthesis and Characterization Laboratory, Advanced Institute of Technology, Universiti Putra Malaysia (UPM), 43400 Serdang, Malaysia; ^3^Wireless and Photonics Network Research (WIPNET), Faculty of Engineering, Universiti Putra Malaysia (UPM), 43400 Serdang, Malaysia; ^4^Department of Civil Engineering, Universiti Putra Malaysia (UPM), Malaysia, 43400 Serdang, Malaysia

## Abstract

The particle size, morphology, and stability of Ag-NPs were investigated in the present study. A Q-Switched Nd: YAG pulsed laser (*λ* = 532 nm, 360 mJ/pulse) was used for ablation of a pure Ag plate for 30 min to prepare Ag-NPs in the organic compound such as ethylene glycol (EG) and biopolymer such as chitosan. The media (EG, chitosan) permitted the making of NPs with well dispersed and average size of Ag-NPs in EG is about 22 nm and in chitosan is about 10 nm in spherical form. Particle size, morphology, and stability of NPs were compared with distilled water as a reference. The stability of the samples was studied by measuring UV-visible absorption spectra of samples after one month. The result indicated that the formation efficiency of NPs in chitosan was higher than other media and NPs in chitosan solution were more stable than other media during one month storage. This method for synthesis of silver NPs could be as a green method due to its environmentally friendly nature.

## 1. Introduction

Recently, a lot of researches interested in metallic nanostructure materials have come up due to their unusual properties which are different from their bulk materials such as their electronic, optical, magnetic, and chemical properties [1]. Due to these properties the main attraction for scientists is applications of these nanoparticles (NPs) in technology. For instance antibacterial and nano-composite are numbers of the most crucial applications of silver nano particles (Ag-NPS) [2–4]. Hence, various NPs or nanocomposite materials have been investigated for their antimicrobial activity as growth inhibitors [5]. A lot of methods such as chemical methods, sol gel, sonochemial method, and laser ablation (LA) were used to prepare the Ag-NPs [6–9].

Nevertheless, such chemical reduction method is not recommended since the chemicals are highly reactive and known to pose a potential environmental hazard and biological risks. Instead, a variety of green technologies for the preparation of Ag-NPs have been developed [10]. Newly, plasma assisted methods based in LA. The advantage of LA compared to chemical synthesis is the simplicity of the procedure and also absence of chemical reagents in solution. Furthermore, the laser pulse has appeared to be more flexible and promising technique for the reason that it is proficient to ablate different type of materials such as metals, ceramic and polymer considers the ultra-high energy density. In LA, the control over the growth process was provided by manipulating the process parameters like irradiation time, duration, energy density, wavelength, and so forth [11]. LA technique is based on ablating a solid target in a gas or a liquid environment. The more effective collection of synthesized NPs can be achieved by LA in a liquid phase. The most important features of the LA technique have been studied by many researchers [12, 13], due to the aqueous media which is highly effective on the particle size and stability. Recently, using an organic solvent as a stabilizer for synthesis of NPs has been investigated [14, 15]. Among all organic solvents, ethylene glycol (EG) (HOCH_2_CH_2_OH) got more attraction due to widespread chemical and physical properties and applications. EG is a colorless, practically odorless, with relatively low-volatility, and is hygroscopic liquid with low viscosity. Indeed, it is completely miscible with water, many organic liquids, and many polar solvents (e.g., alcohols, glycol, ethers, and acetone) and vaguely soluble in nonpolar solvent such as toluene, benzene, and chloroform [16–19].

On the other hand, among the natural polymers, chitosan is the second most naturally abundant polysaccharide that can be easily isolated from crustacean shell. Since chitosan is nontoxic and has been approved by Food and Drug Administration [20], in this case, the dispersed Ag-NPs in chitosan solution does not necessarily need to be separated and purified [21].

In this work, we preformed LA silver plate in EG and chitosan to prepare Ag-NPs. In the LA process, we hypothesized that the Ag-NPs size decreases. Conversely, the stability of NPs increases in these media to compare with in distilled water (DW).

## 2. Experiment

**Figure 1:**
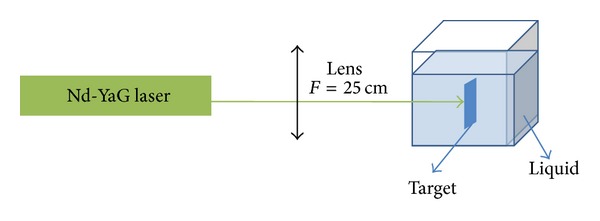
The LA setup for the colloidal NPs production.

The schematic diagram of the LA experimental setup is indicated in Figure 1. A pulsed Q-Switched Nd: YAG laser (SL400/SL800 system) with pulse duration of 10 ns and 30 Hz repetition rate at a second harmonic wavelength (532 nm) was applied to prepare the Ag-NPs. A silver plate (99.99% purity; Sigma Aldrich) was located in cubic cell containing 10 mL of EG and chitosan that the appropriate amounts of 0.2 g chitosan were dissolved separately 100 mL in distilled water at 60°C and stirred for 1 hr. Prior to ablation, the silver plate was cleaned by using an ultrasonic bath for 30 min, and it was immersed in the solution. The solution was magnetically stirred at room temperature during the ablation process to disperse the produced NPs. The laser output power 35 mJ/pulse was measured by the optical power detector. The laser beam was focused on the silver target by a 25 cm focal length lens. The ablation was carried out at room temperature for 30 min. The same experiment has repeated for DW as the solvent as a making reference to measure. The prepared samples have been characterized using a UV-visible, double beam photospectrometer (UV-1650 PC, Shimadzu) with 1 cm optical path cell, transmission electron microscopy (TEM, Hitachi H-7100; Hitachi) at 120 KV accelerating voltages, and Fourier transform infrared (FT-IR) spectrometer (1650; Perkin Elmer, Waltham, MA).

## 3. Results and Discussion

**Figure 2:**
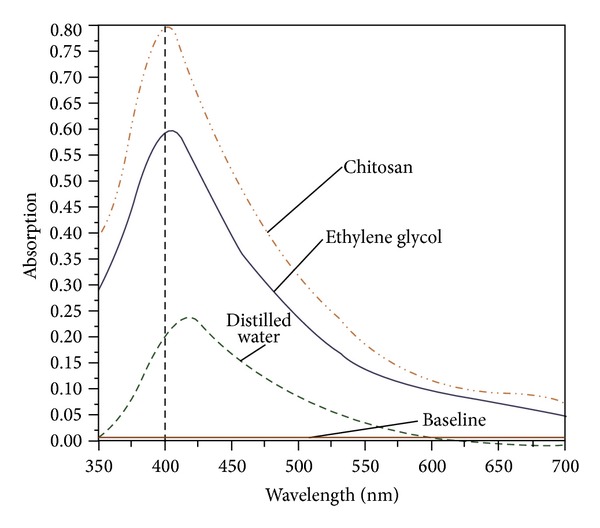
UV-visible absorption spectra of Ag-NPs prepared for 30 min ablation times in EG, chitosan, and DW.

**Figure 3:**
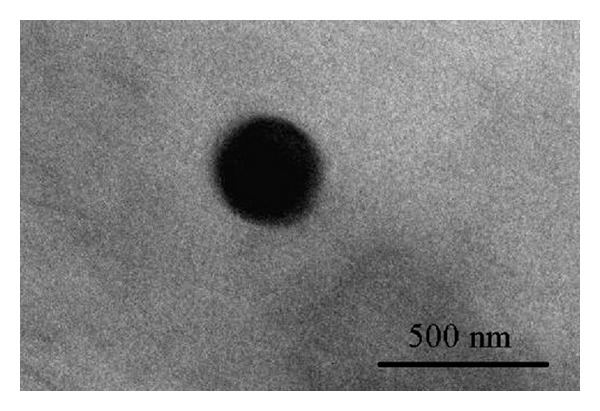
TEM images of Ag-NPs in ethylene glycol.

**Figure 4:**
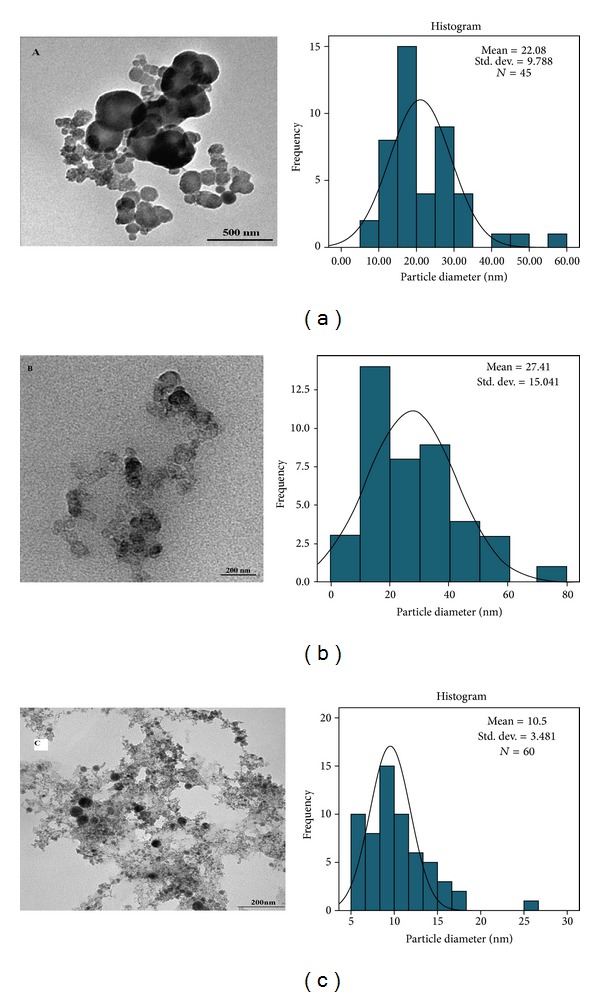
TEM image and typical of statistical graph for Ag-NPs produced in (a) EG, (b) in DW, and (c) in chitosan under 30 min ablation times in temperature room.

The solutions are observed to change color from its colorless and transparent form to slightly yellowish one after a few minutes during the ablation of the silver plate. The dark yellow will be achieved for higher concentration. This was also confirmed by UV-visible absorption spectra.  Figure 2 indicates the optical absorption spectra of the solutions containing Ag-NPs. The peak at 400 nm is the signature of plasmon peak of Ag-NPs, which confirmed the Ag-NPs, and was formed inside the aqueous media. To compare with DW the peak intensity increase [22] and had a blue shift toward high energy, which shows an increase in the formation efficiency of the NPs and indicates a reduction of particle size following the Mie theory [23]. Furthermore, the spectrum peaks at this wavelength signify that the NPs in the solutions are spherical which is confirmed by TEM results shown in Figures 3 and 4 [24]. From Figure 2, the intensity of absorption peak at 400 nm was increased that can conclude the number of generated NPs was also increased. The increase of formation efficiency is due to the increment of the density and viscosity of solvent.

In contrast, in Figure 4(a) size reduction can be explained by the interaction between the EG molecules and laser products. In this step silver atoms interact with EG molecules; therefore the initial silver particles are formed because of this interatomic interaction. The mechanism of protecting particles from aggregation by EG can be explained by the hydroxyl group. In the fact of competition, the EG molecules now can absorb particles and prevent them from aggregation and growth [25]. On the other hand, Figure 4(c) shows Ag-NPs in chitosan and their corresponding size distribution that the obtained mean particle size is about 10.5 nm. Referring to Figure 4(c), TEM analysis also indicates that Ag-NPs are well dispersed with spherical morphology and there is no evidence of agglomeration.

Figures 3 and 4 depict the TEM images that Figure 3 indicates the spherical shape of Ag-NPs [26]. This type of NPs shape is suitable for drug loading and most biological applications, such as antibacterial properties [27]. Measurements of the mean size of Ag-NPs are 22.08 nm in EG and are about 10.5 nm in chitosan and the mean size of Ag-NPs is 27.41 nm in DW which ablation time for all cases completed in 30 min. Indeed, confirms the observation of particle size decrement with respect to the media as shown in Table 1. The formation efficiency of NPs in chitosan is higher than in EG and that in EG is higher than in DW (chitosan > EG > DW) due to the confinement on the Ag plate produced by ablation and it becomes stronger with the increase of solvent density and viscosity. In this case, chitosan has higher density and viscosity than other solutions; for these reasons the effect of size decrement for Ag-NPs is observed. The plasma is generated close to the plate with high pressure, which is confined surface, and it can etch the surface to make NPs [28, 29]. The procedure called secondary ablation [30] can improve efficiency of the formation of Ag-NPs. The other interesting point that can be observed from Figure 2 is that, by increasing the efficiency of formation of NPs due to increase of the density and viscosity of aqueous media, the plasmon peak also shifts toward higher energies. According to Mie's theory, the blue shift of spectra shows that the particle sizes were decreased.

**Figure 5:**
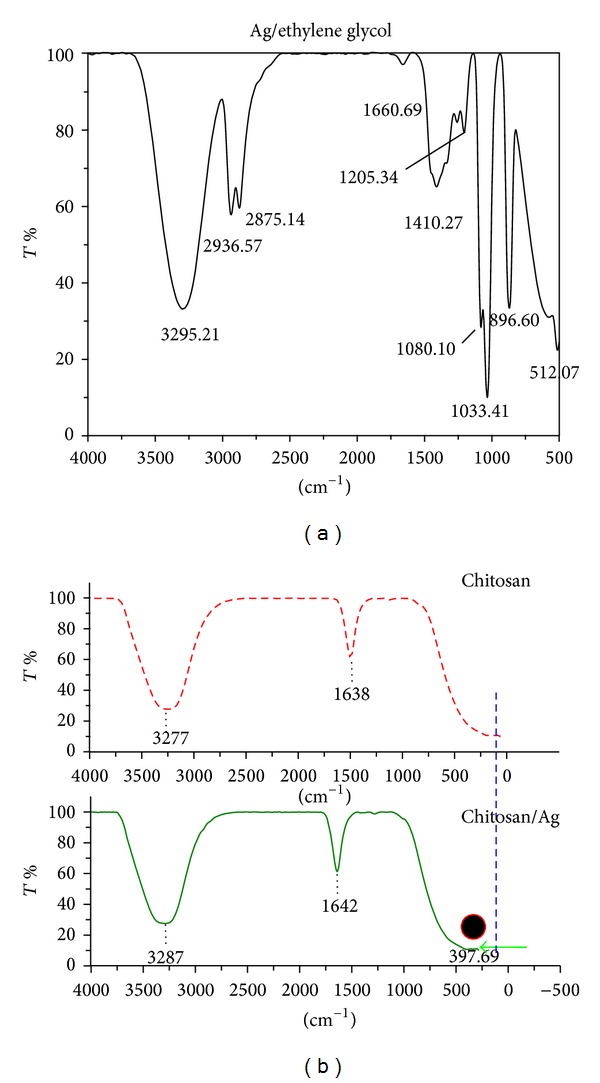
FT-IR spectra of Ag-NPs in (a) EG and (b) chitosan using LA.

**Figure 6:**
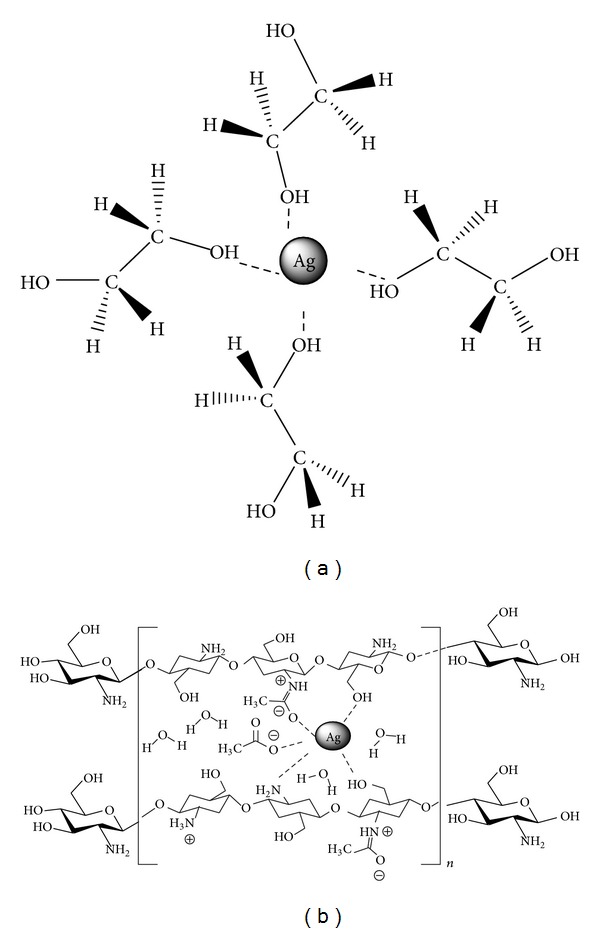
Mechanism of capping Ag-NPs by (a) ethylene glycol and (b) chitosan.

Infrared spectroscopy, IR radiation is passed through a sample. Some of the infrared radiation is absorbed by the sample and some of it is passed through (transmitted). The resulting spectrum represents the molecular absorption and transmission, creating a molecular fingerprint of the sample. FT-IR spectra in Figure 5 confirm the formation of Ag NPs inside the solutions. The spectrum in Figure 5(a) indicates absorption peak at about 512.07 cm^−1^ which is a signature of Ag-NPs bonding with oxygen from hydroxyl groups [31] and Figure 6 shows the mechanism of protecting particles from aggregation by this stabilizer. The absorption peaks at about 3296.48, 1660.69 cm^−1^ which presented O–H groups. Furthermore, the peaks in 2936.57, 2875.14 cm^−1^ were assigned to symmetric and asymmetric of C–H_2_ stretching [32] the band at 1410.27 cm^−1^ corresponds to C–H_2_ bending. However, the peak at 1205.34 cm^−1^ corresponds to C–H stretching [31] and the peak at 1080.10 cm^−1^ indicates the bending of C–C–O [32].

In competition, the chitosan molecules can absorb particles and prevent their aggregation and growth [33]. The mechanism of protecting particles from aggregation by chitosan can be explained by those nitrogen atoms of amino group in chitosan which hold a free electron doublet that is responsible for the uptake of NPs by chelating mechanism [34]. Some researchers have attributed a key role to the amine in the Ag+ reduction due to its decrease in the potential of Ag+/Ag (EAg+/Ag) promoting the reaction [32]. However, there is still no substantial evidence that confirms this assumption.

**Figure 7:**
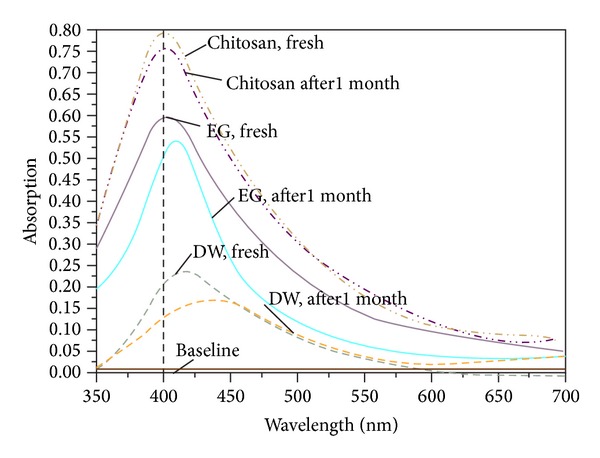
UV-visible absorption spectra of Ag-NPs in EG, DW, and chitosan for freshly prepared and after 1 month.

As well, the UV-visible spectra of the Ag-NPs in EG, chitosan, and the water were measured after 1 month to investigate the capability of fluids as a stabilizer. The absorption spectrum in Figure 7 does not show a significant change in the fresh sample compared to old sample in chitosan, but a large reduction can be seen in EG and water. It shows the Ag-NPs in chitosan were stable. Moreover, there is a red shift which indicates some agglomeration of the Ag-NPs in EG and water; hence the vague descent in the absorption intensity is due to the slight sedimentation of the larger particles [35].

## 4. Conclusions

The synthesis of Ag-NPs in chitosan and EG using a LA technique was presented as a simple and green method. For a similar laser ablation time (30 min) the obtained particle size is smaller (10.5 nm) for chitosan than (22.08 nm) for EG and DW (27.41 nm). The particle size reduced with the effective solvent rather than with pure water. The obtained NPs are stable for quite a long time in chitosan because it controls the particle size and thus prevents agglomeration between the ablated NPs.

## Figures and Tables

**Table 1:** Particle size of Ag-NPs with their standard deviation for particle of size (nm) in ethylene glycol and distilled water.

Ablation time (min)	Media	Particle size (nm)	Standard deviation (nm)
30	Ethylene glycol	22.08	9.788
30	Distilled water	27.41	15.041
30	Chitosan	10.50	3.481
